# Hepatectomy using a combination of extrafascial extrahepatic (Takasaki approach) and extrafascial intrahepatic pedicle approaches (Ton That Tung approach)

**DOI:** 10.1093/jscr/rjab419

**Published:** 2021-10-04

**Authors:** Quyet Van Ha, Toan Huy Nguyen, Huong Van Nguyen, Xuan Anh Le, Kinh Huy Tran

**Affiliations:** Department of General Surgery, Ha Noi Medical University, Hanoi, Vietnam; Department of General Surgery, Nghe An General Hospital, Nghe An Province, Vietnam; Department of General Surgery, Nghe An General Hospital, Nghe An Province, Vietnam; Department of General Surgery, Nghe An General Hospital, Nghe An Province, Vietnam; Department of General Surgery, Nghe An General Hospital, Nghe An Province, Vietnam

## Abstract

Selective pedicle control and anatomical liver resection are considered standard techniques in hepatectomy for hepatocellular carcinoma. In 1963, Ton That Tung made significant improvements in hepatectomy techniques with the principle of locating and ligation of Glissonean pedicle in the liver parenchyma based on precise knowledge of vascular and biliary anatomy (Tung TT, Quang ND. A new technique for operating on the liver. *Lancet* 1963;**281**:192–3). In 1986, the extrafascial Glissonean dissection was first introduced by Takasaki in 1986. This is a simple and safe technique that helps to identify the exact borders between liver sections for anatomic liver resection (Takasaki K. Glissonean pedicle transection method for hepatic resection: a new concept of liver segmentation. *J Hepatobiliary Pancreat Surg* 1998;**5**:286–91). The combination of two techniques helps minimize complications, reduce ischemic time of future liver remnant, intraoperative blood loss and avoid migration of cancer cells into other segments.

## INTRODUCTION

According to the Global Cancer Observatory database in 2020, it was estimated that there were 26 418 new liver cancer cases in the world [1]. Recently, there have been several modifications in the patient selection and improvements of surgical techniques which help reduce complications, recurrence and optimize the prognosis of patients with hepatocellular carcinoma.

## TECHNIQUE

A modified technique of hepatectomy was used which combines the advantages of Ton That Tung and Takasaki’s approaches [2,3].

**Figure 1:**
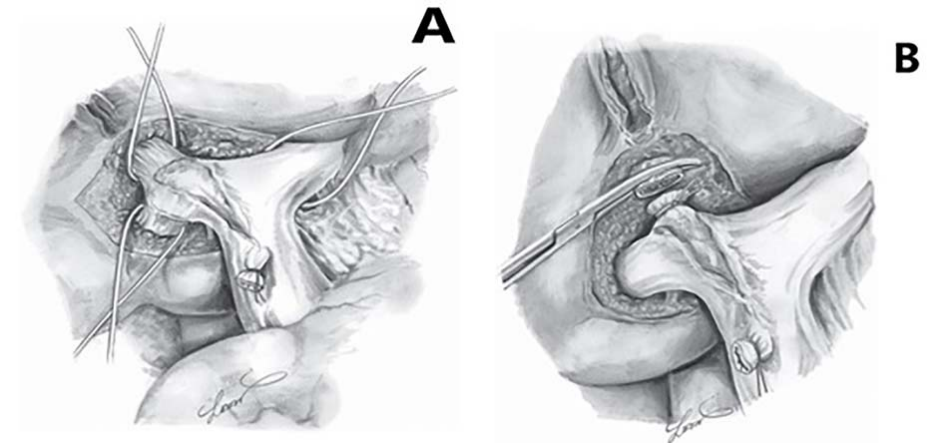
(**A**) Control of extrahepatic Glissonean pedicles using the Takasaki approach [4]. (**B**) Divide the Glissonean pedicle before parenchymal transection.

Step 1: Extrahepatic extrafascial Glissonean dissection is performed using Takasaki’s approach: After cholecystectomy, the lesser omentum is opened to expose the liver hilum (Fig. 1). Dissection at the bifurcation is carried out with attention to ligate all small branches to Segment 4 to avoid the bile leak and bleeding. Three main Glissonean pedicles (left, right anterior, right posterior) are exposed.

**Figure 2:**
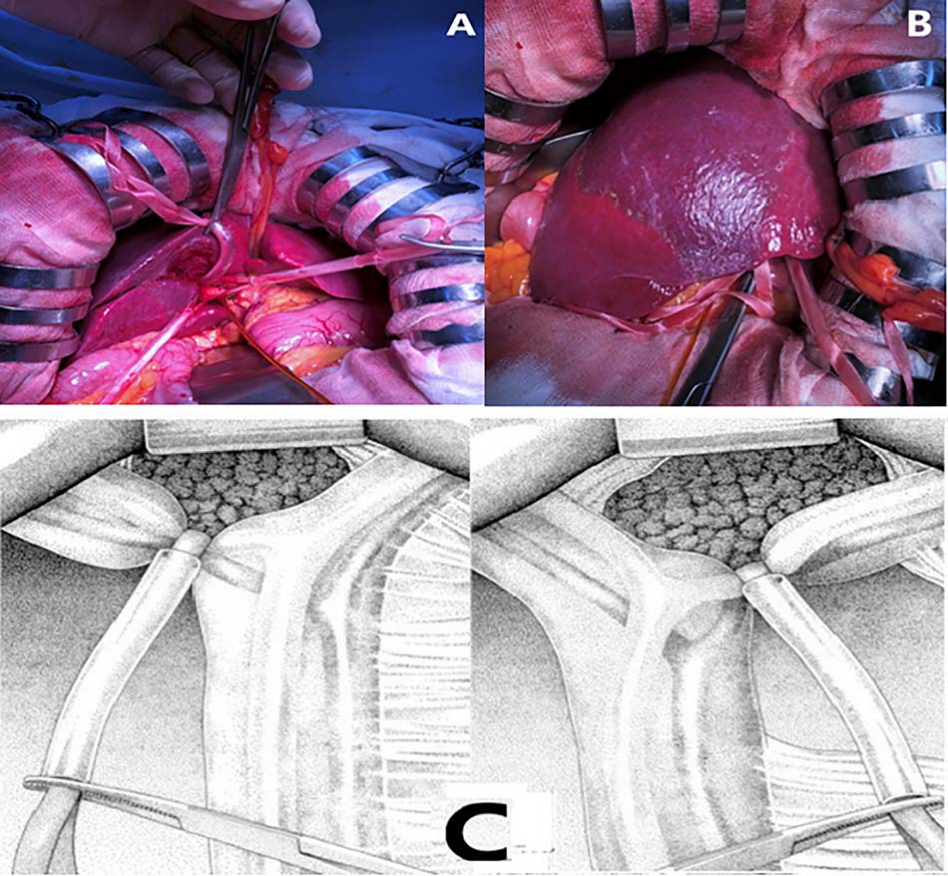
(**A**), (**C**). Temporary occlusion of the extrahepatic Glissonean pedicle using tape and silicone tube. (**B**) Demarcation line clearly observed on the surface after clamping.

- The pedicle of the to-be-resected section is taped and temporarily occluded instead of early division before parenchymal transection as in the original Takasaki’s approach (Fig. 2).

**Figure 3:**
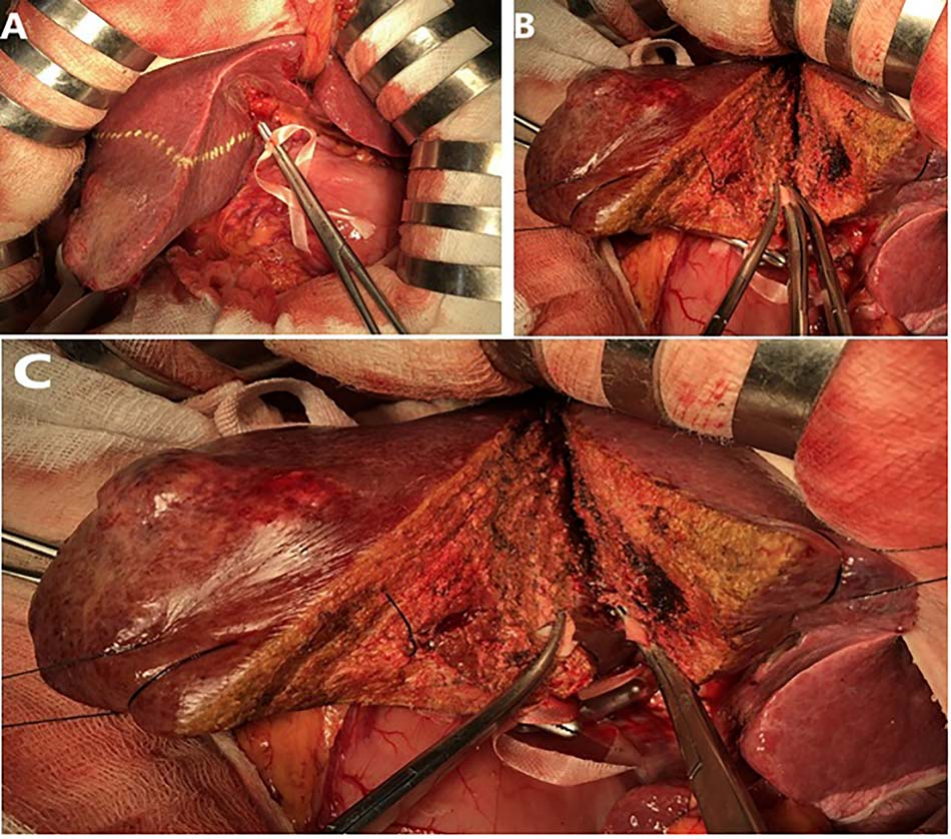
Glissonean pedicles are divided inside the parenchymal (suture–ligation) after being exposed by clamp-crush technique.

Step 2: Parenchymal transection using Ton That Tung approach: transection of the parenchymal is done followed by intrahepatic division of Glissonean pedicles. This modified technique help avoid complications due to liver anatomical variations (Fig. 3).

- Demarcation line is clearly marked on the surface after color change following clamping of the Glissonean pedicle. Transection is then proceeded following the demarcation line.
- Transection is performed by clamp-crush technique (Kelly hemostatic forceps, Harmonic scalpel). Vascular and biliary structures are dissected and ligated at the resection line [5].

Hepatic veins are also divided inside the liver parenchyma. The hepatic veins are then sutured and ligated using Prolene 4.0 suture.

**Figure 4:**
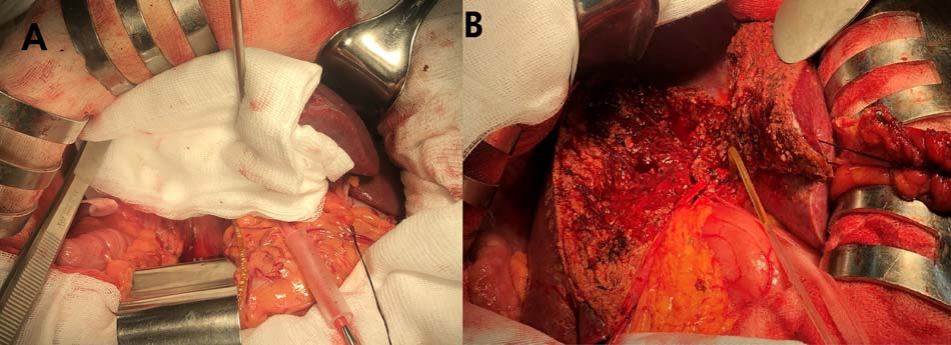
Bile leak control using (**A**) abdominal gauze pads. (**B**) Methylene blue injection via cystic duct stump.

Step 3. Bile leak test at the resection margin using two methods (Fig. 4):

- A white abdominal gauze pad is firmly applied on the cut surface during 5–10 minutes. Bile leak signified by yellow spots on the gauze pad is then controlled.
- Methylene blue injection via cystic duct stump.

## DISCUSSION

Hepatectomy for hepatocellular carcinoma is a technically demanding procedure for provincial hospital such as Nghe An General Hospital due to the lack of sophisticated surgical instruments for hepatectomy [cavitron ultrasonic surgical aspirator (CUSA), intraoperative ultrasound…]. Therefore, our modified approach can combine the advantages of different methods, resulting in optimal results to patients and reduced complications with minimal resources.

From February 2017 to July 2021, we performed hepatectomy for hepatocellular carcinoma in 83 patients using this technique with good results: mean operative duration 159.7 ± 53.12 (65–315) minute, mean blood loss volume 278.82 ± 126.17 mL for major hepatectomy and 237.41 ± 150.33 mL for minor hepatectomy. There was no biliary and vascular injuries related to liver anatomical variations.

## CONCLUSIONS

This modified technique is proved to reduce the complication rates related to the liver anatomical variation at limited resource institutions without sophisticated instruments (intraoperative ultrasound, CUSA …).

## AUTHORS’ CONTRIBUTIONS

Dr Quyet Van Ha: literature review, review and editing.

Dr Toan Huy Nguyen: study concept, primary surgeon, data collection and writing the manuscript.

Dr Huong Van Nguyen: literature review and final review.

Dr Xuan Le Anh: final review.

Dr Kinh Huy Tran: final review.

All authors read and approved the final manuscript.
